# Multi‐Physical Field Modulated P‐Bit Device Based on VO_2_ Thin Film

**DOI:** 10.1002/advs.202524248

**Published:** 2026-02-16

**Authors:** Bowen Sun, Jianjun Li, Ting Zhou, Jinglin Zhu, Meiling Liu, Zhihan Lin, Chang Wang, Chengyu Li, Yingxue Chen, Xiaokun Huo, Chongwen Zou

**Affiliations:** ^1^ National Synchrotron Radiation Laboratory, School of Nuclear Science and Technology University of Science and Technology of China Hefei Anhui P. R. China

**Keywords:** mott oscillator, phase‐change‐material (PCM), probability bit (P‐bit), vanadium dioxide

## Abstract

Probabilistic computation using probability bits (P‐bits) is highly effective for combinatorial optimization problems such as integer factorization because of its fast search ability. However, implementing P‐bits with traditional complementary metal‐oxide‐semiconductor (CMOS) technology usually needs external noise for randomness, which complicates fabrication and system integration. To overcome these challenges, we have proposed a VO_2_‐based P‐bit device where synergistic multi‐physical field modulation (electric, thermal, optical) enables real‐time tunability of randomness—an obvious advance beyond previous P‐bits, which rely primarily on single‐field control. This P‐bit provides excellent durability and inherent randomness, with output probability that can be adjusted via multi‐physical field modulation. Besides introducing a new phase‐change material‐based device approach for high‐performance P‐bits, this study also demonstrates a synergistic multi‐physical field modulation strategy that opens new opportunities for neuromorphic device applications.

## Graphical abstract

We have proposed a VO_2_‐based P‐bit device where synergistic multi‐physical field modulation enables real‐time tunability of randomness. Besides introducing a new phase‐change material‐based device approach for high‐performance P‐bits, this study also demonstrates a synergistic multi‐physical field modulation strategy that opens new opportunities for neuromorphic device applications.

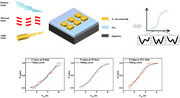

## 1 Introduction

The probability bit (P‐bit), an entity capable of fluctuating between the two Boolean states of 0 and 1, serves as the core unit of probabilistic computing [1, 2]. Unlike traditional deterministic computing architectures, P‐bits provide hardware‐level true random sources that serve as physical substrates for probabilistic computation, including Bayesian inference [3, 4], annealing‐based approaches to combinatorial optimization [5, 6], and stochastic spiking neuron models in spiking neural networks (SNN) [7, 8, 9]. Experimental evidence shows that such stochasticity effectively prevents algorithms from becoming trapped in local optima, thereby enhancing the fault tolerance and energy efficiency of complex systems [10]. In recent years, P‐bit implementations based on true random sources such as complementary metal‐oxide‐semiconductor (CMOS) circuits [11, 12], magnetic tunnel junctions [5, 13], and the spin‐orbit effect [7, 14] have made significant progress. However, intrinsic limitations like challenges in controllability, limited randomness sources, and scalability issues continue to hinder large‐scale integration and practical use. Notably, most existing devices depend on external noise injection [15, 16], which inherently limits their randomness and fails to meet the requirements for robust true random sources in neuromorphic systems. For instance, in traditional SNN, the deterministic encoding of the input layer generates predictable spike sequences, which inherently limit their ability to capture the intrinsic randomness of biological neurons. In biological systems, neuronal discharges exhibit probabilistic characteristics even when exposed to identical stimuli [9]. These requirements align closely with the core functionality of P‐bit devices—as physically meaningful true random entropy sources, they possess randomly discharged characteristics with well‐defined probability distributions. Therefore, developing new P‐bit devices that harness intrinsic physical randomness while maintaining structural simplicity and tunability has become a key goal for overcoming the bottlenecks of the von Neumann architecture.

Vanadium dioxide (VO_2_), as a strongly correlated material, has become a promising candidate for neuromorphic device fabrication due to its ultrafast metal–insulator transition (MIT) near room temperature (∼68°C) [17, 18, 19, 20]. This transition involves a cooperative reconstruction of electronic states and crystal structure, enabling resistive switching on a nanosecond scale under external stimuli (electrical, thermal, or optical) [21]. More importantly, the intrinsic stochasticity of domain wall dynamics during the VO_2_ phase transition results from thermodynamic fluctuations, providing a natural physical basis for true random P‐bits [22, 23]. Despite this potential, the practical use of VO_2_ remains limited by two main challenges: fabrication issues, as the narrow growth window for VO_2_ phases makes it difficult to produce stable, high‐quality thin films; and complex regulation, since the cooperative mechanisms of multi‐physical field (electrical, thermal, optical) on the phase transition are not yet fully understood, hindering precise probabilistic device design.

To address these challenges, in this study, we have systematically explored the effects of multi‐physical field modulated VO_2_ phase transition and the related random P‐bits device. High‐quality VO_2_ thin films were epitaxially grown on C‐cut sapphire substrates using oxygen‐assisted molecular beam epitaxy (O‐MBE), achieving sufficient stability to withstand >10^6^ cycles of resistor switching tests. Additionally, the cooperative regulation mechanism of multi‐physical field was elucidated: environmental thermal field and infrared laser irradiation (1550 nm) were shown to reduce the resistance of the insulating state and increase Joule‐heat accumulation, thereby lowering the threshold (V_th_) and holding (V_hold_) voltage as a function of temperature and optical power density, adding a new dimension for probabilistic control. Previous studies on VO_2_‐based P‐bit have often utilized Pump–Probe pulsed detection of resistance states to generate random sequences, thus necessitating reliance on Pump pulses for resetting. In this work, we innovatively employ a Pearson–Anson circuit to directly output random sequences under constant voltage. To the best of our knowledge, this is the first reported P‐bit device based on a VO_2_ Mott oscillator that allows real‐time tunability of probability modulated by multi‐physical fields.

## 2 Results

### 2.1 VO_2_ Thin Film Characterizations

**FIGURE 1:**
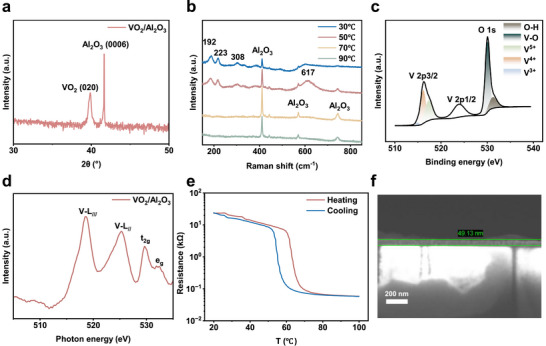
Characterizations of VO_2_/sapphire thin film. (a) XRD analysis of the film sample. (b) Temperature‐dependent Raman spectroscopy of VO_2_ thin film. (c) and (d) The XPS and XANES spectra for the film sample. (e) The *R–T* curve of the VO_2_ thin film during heating and cooling processes. (f) SEM cross‐section of VO_2_ thin film deposited on sapphire. The thickness of the VO_2_ thin film is approximately 50 nm.

VO_2_ thin films were grown on C‐cut sapphire substrates using O‐MBE, with characterizations summarized in Figure 1. The XRD test for the film sample (Figure 1a) showed the preferred monoclinic VO_2_ (020) orientation with the sharp XRD peak at 39.8°. A temperature‐dependent Raman test was also conducted to examine the samples. Figure 1b shows the featured Raman peaks at 192, 223, 308, and 617 cm^−^^1^ for the deposited monoclinic VO_2_ film at 30 or 50 degrees. While increasing the temperature to 70 or 90 degrees, the Raman peaks fromVO_2_ film disappeared completely, mainly due to the phase transition from monoclinic to rutile R phase, confirming the typical insulator‐metal property of the VO_2_ film sample. To examine chemical states, XPS and XANES tests were performed (Figure 1c,d). In the XPS spectrum (Figure 1c), the V 2p_3_/_2_ peak at 516.3 eV primarily originated from V^4^^+^ (516.2 eV), while a smaller V^5^^+^ peak at 517.4 eV indicated the slight surface oxidation due to the XPS surface sensitivity. The O 1s peak at 530.1 eV corresponds to oxygen in VO_2_, and the 531.3 eV peak to O─H bonding from trace adsorbed oxygen [18]. Synchrotron‐based XANES spectra (Figure 1d) showed distinct V–L edges at 518.6 eV (2p_3_/_2_) and 525.3 eV (2p_1_/_2_), along with clear O‐K edge features [21]. The MIT behavior (Figure 1e) demonstrates a clear resistivity change near 67°C. The SEM cross‐section (Figure 1f) revealed a uniform ∼50 nm film thickness. These results confirm the high quality of the VO_2_ thin films grown by the O‐MBE method. It is worth noting that our primary objective in employing MBE was to demonstrate the application potential of high‐quality single‐crystal VO_2_ films as P‐bits under multi‐physical field control. However, the device fabrication process is fully compatible with polycrystalline techniques, and the grain boundary defects introduced during polycrystalline deposition may further enhance its randomness.

### 2.2 Multi‐Physical Field Modulated Two‐Terminal Planar VO_2_ Device

**FIGURE 2:**
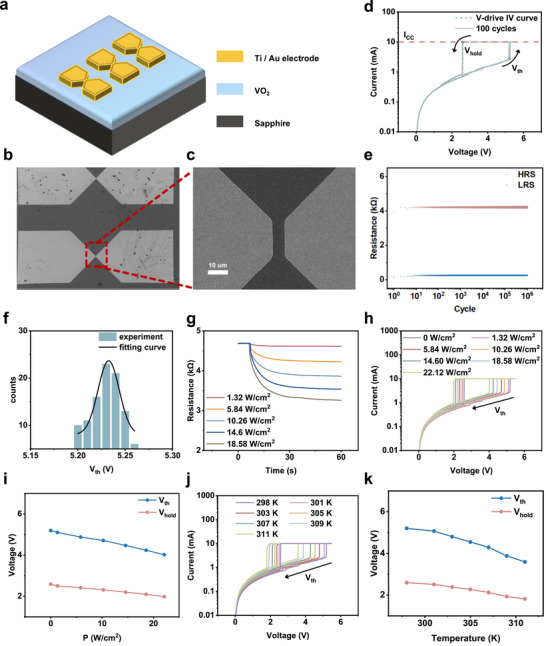
Multi‐physical field response of two‐terminal VO_2_ device. (a) Schematic of the two‐terminal VO_2_ device. (b) and (c) SEM images of the device. The VO_2_ gap is about 5 µm × 18 µm. (d) Current–voltage characteristics of the device repeated over 100 cycles. (e) Distributions of high and low resistance states across 10^6^ cycles. (f) V_th_ distribution derived from IV testing, fitted to a Gaussian distribution. (g) Variable resistance response of the device under laser irradiation (1550 nm) at various power densities. (h) *I–V* curves of the device under laser irradiation with different power densities. (i) V_th_ and V_hold_ curves in (h). (j) The *I–V* curves of the device at different ambient temperatures. (k) V_th_ and V_hold_ curves in (j).

As shown in Figure 2a, two‐terminal planar VO_2_ devices were made using standard fabrication techniques. The SEM image in Figure 2b and the enlarged view in Figure 2c clearly showed the two gold electrodes and the VO_2_ gap, which indicated the gap length and width values were 5 and 18 µm, respectively. The more details were further characterized by Energy Dispersive Spectrometer (EDS) and Atomic Force Microscopy (AFM), shown in Figure S1. For the *I–V* test in Figure 2d, the voltage step was set at 0.01 V with a 10 mA compliance current to avoid damage. Results showed that the two‐terminal VO_2_ device showed the typical sharp‐jumping/dropping current with the distinct V_th_ and V_hold_ points during the *I–V* test. Specifically, when the applied voltage exceeded the V_th_ value, the device switched from a high resistance state (HRS) to a low resistance state (LRS), while when it dropped below V_hold_, it reverted to HRS. These typical *I–V* features basically originated from the metal‐insulator transition of VO_2_ layer, while not from the normal defect‐driven memristor caused by ion migration [1] or redox reactions [24]. The resistive switching states during 10^6^ cycles (Figure 2e) were quite stable, confirmed the high crystallinity and durability of the two‐terminal device based on the crystal VO_2_ film prepared by O‐MBE. Figure 2f showed the threshold Vth distribution extracted from 100 cyles *I–V* tests performed on a single device in Figure 2d. The Vth values showed a Gaussian distribution reflecting the stochastic characteristic of electric field driven transitions. These stochastic characteristics were mainly due to the intrinsic domain flipping [22], which formed the basis for VO_2_‐based P‐bit devices [25].

In fact, the electric feature of the two‐terminal VO_2_ device was sensitive to the external multi‐physical field, and the modulation effects had been an active research point [21]. Here, we examined the electric response upon the 1550 nm pulsed laser radiation on the VO_2_ gap. By applying a 10 µA current, the resistance was monitored under the laser pulse radiation with various optical powers (Figure 2g). It was observed that the insulating resistance decreased as power increased, consistent with previous optical studies [20]. The further *I–V* tests under laser irradiation (Figure 2h) still showed the typical hysteresis behavior, while the systematic reductions in V_th_ and V_hold_ with increasing optical power (Figure 2i) were observed, confirming the electrical–optical synergic modulation effect. Although the deep understanding of the origination of electrically triggered VO_2_ transitions is still debated, the dominant view links switching to Joule heating [26] and electric field effect [27].

It should be pointed out that in our experiments, the two‐terminal VO_2_ device was fully volatile, which indicated that no ion migration processes occurred due to the electroforming or filament dynamics [28]. The decrease in V_th_ value under infrared laser irradiation could be explained by the laser‐induced local heating effect. To further examine the thermal–electrical coupling, I–V curves were recorded at different ambient temperatures (Figure 2j,k). Similar to optical stimulation, the higher temperature conditions also reduced both the V_th_ and V_hold_ values. These findings showed that multi‐physical field coupling was an effective way to adjust VO_2_ phase transition dynamics [21].

### 2.3 P‐Bit Device Based on VO_2_ Mott Oscillator

**FIGURE 3:**
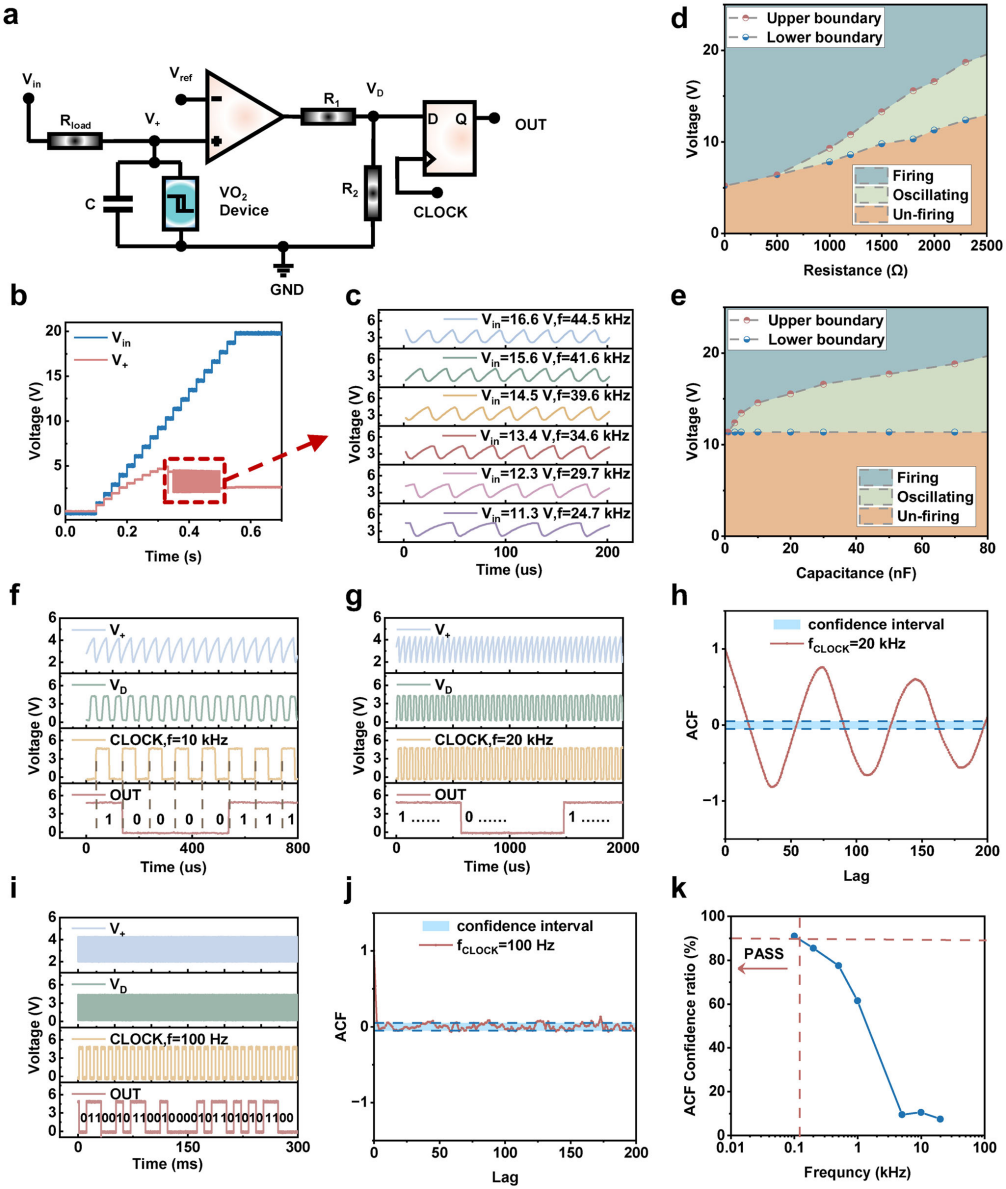
The performance of the VO_2_ film‐based P‐bit device. (a) The device scheme. (b) Output of VO_2_ Mott oscillator under voltage input. (c) Detailed oscillation waveform in (b), where the oscillation frequency increases with the rising input voltage. (d) and (e) Phase diagrams of the VO_2_ Mott oscillator corresponding to different series resistances and parallel capacitances. (f) The device performance is recorded. When the rising edge of CLOCK occurs, the output updates to the current value of V_D_. (g) and (i) Bit outputs of the circuit when the CLOCK frequency is 20 kHz and 100 Hz, respectively. (h) and (j) Autocorrelation coefficient curves of the bit trains at CLOCK frequencies of 20 kHz and 100 Hz, respectively, with a 95% confidence interval. (k) Confidence level of the P‐bit output ACF under different clock frequencies. At high frequencies, the bit trains exhibit strong autocorrelation, while at low frequencies, they display high randomness.

To investigate the inherent randomness of electrically induced phase transitions in two‐terminal VO_2_ devices, we encapsulated the fabricated device and assembled the circuit shown in Figure 3a on a breadboard, creating a P‐bit device based on a VO_2_ Mott oscillator [29]. Basically, in the circuit, a VO_2_ Mott oscillator built with a Pearson–Anson circuit [30, 31] would generate a self‐sustained oscillation signal V_+_. Then, a comparator compared the V_+_ value with a reference voltage V_ref_, producing a rectangular wave signal V_D_ of the same frequency. Finally, a D‐type flip‐flop would output the instantaneous value of V_D_ at each rising edge of an external clock signal (CLOCK). The physical layout was shown in Figure S2. Figure 3b displayed the oscillator response: at low V_in_, the VO_2_ device would remain in the HRS, corresponding to the unfiring region. As V_in_ increased and triggered the MIT, the device would oscillate between the HRS and LRS states, forming the Oscillating region. At higher V_in_, the device kept at the LRS state, entering the Firing region. Figure 3c depicts the oscillation waveform, where capacitor charging and discharging would cause repeated switching. The oscillation waveform shown in Figure 3C consists of multiple electrical excitation cycles. The external capacitance and resistance jointly determined the charging and discharging time constants of the oscillation, while the V_th_ of VO_2_ determined the starting points of these charging and discharging cycles. The oscillation frequency was increased with V_in_, while the duration of the rising edge decreased [32].

Stable and long‐term oscillation output in the device was essential for the practical application. Therefore, we examined the oscillator under various circuit conditions by adjusting the divider resistors and parallel capacitors in the Pearson–Anson circuit. According to the resulting oscillation waveforms (Figures S3, and S4), it was able to create the phase diagrams (Figure 3d,e) for the prepared VO_2_ Mott oscillator, demonstrating that the state depended on both the circuit parameters and input voltage. From the phase diagram, it was clear that different configurations would produce the Unfiring, Oscillating, or Firing regimes, respectively. Then for the subsequent experiments, a stable operating point of 12 V, 2 kΩ, and 50 nF within the Oscillating region was selected. Figure 3f illustrates the bit generation process at V_ref_ = 3.5 V. During stable oscillation, the comparator–divider circuit converted the V_+_ into V_D_, which was then supplied to the D‐type flip‐flop. Controlled by the CLOCK signal, the output was updated to the instantaneous V_D_ value at each rising edge. The number of generated bits depended on the clock frequency.

A key property of a P‐bit was its random fluctuation between Boolean “0” and “1” [5, 33]. Previous studies always linked this randomness to the thermal fluctuations in Mott devices [34, 35]. To determine whether this stochasticity would affect the bit output, we varied the CLOCK frequency to change the fluctuation accumulation time. Figure 3g,i showed the outputs at 20 kHz and 100 Hz, respectively. At 20 kHz, the sequence exhibited long runs of identical bits, while at 100 Hz, it fluctuated randomly. Autocorrelation functions (ACF) were calculated from 1000‐bit sequences (Figure 3h,j). At 20 kHz, strong correlations were observed, whereas at 100 Hz, nearly all points fell within the 95% confidence interval, indicating high randomness performance. Thus, it was clear that lower frequencies allowed longer accumulation of thermal fluctuations, which enhanced the stochasticity [25].

We further assessed the outputs over a wider frequency range (Figures S5 and S6). Figure 3k displayed the ACF confidence levels versus CLOCK frequency, measured by the proportion of lags within the 95% interval. At high frequencies, outputs were strongly correlated, while at low frequencies, they showed weak correlation and high randomness. Only when the CLOCK frequency was below approximately 10^2^ Hz (confidence around 90%), the output could be considered as the valid P‐bit [35].

### 2.4 Multi‐Physical Field Response of P‐Bit Device

**FIGURE 4:**
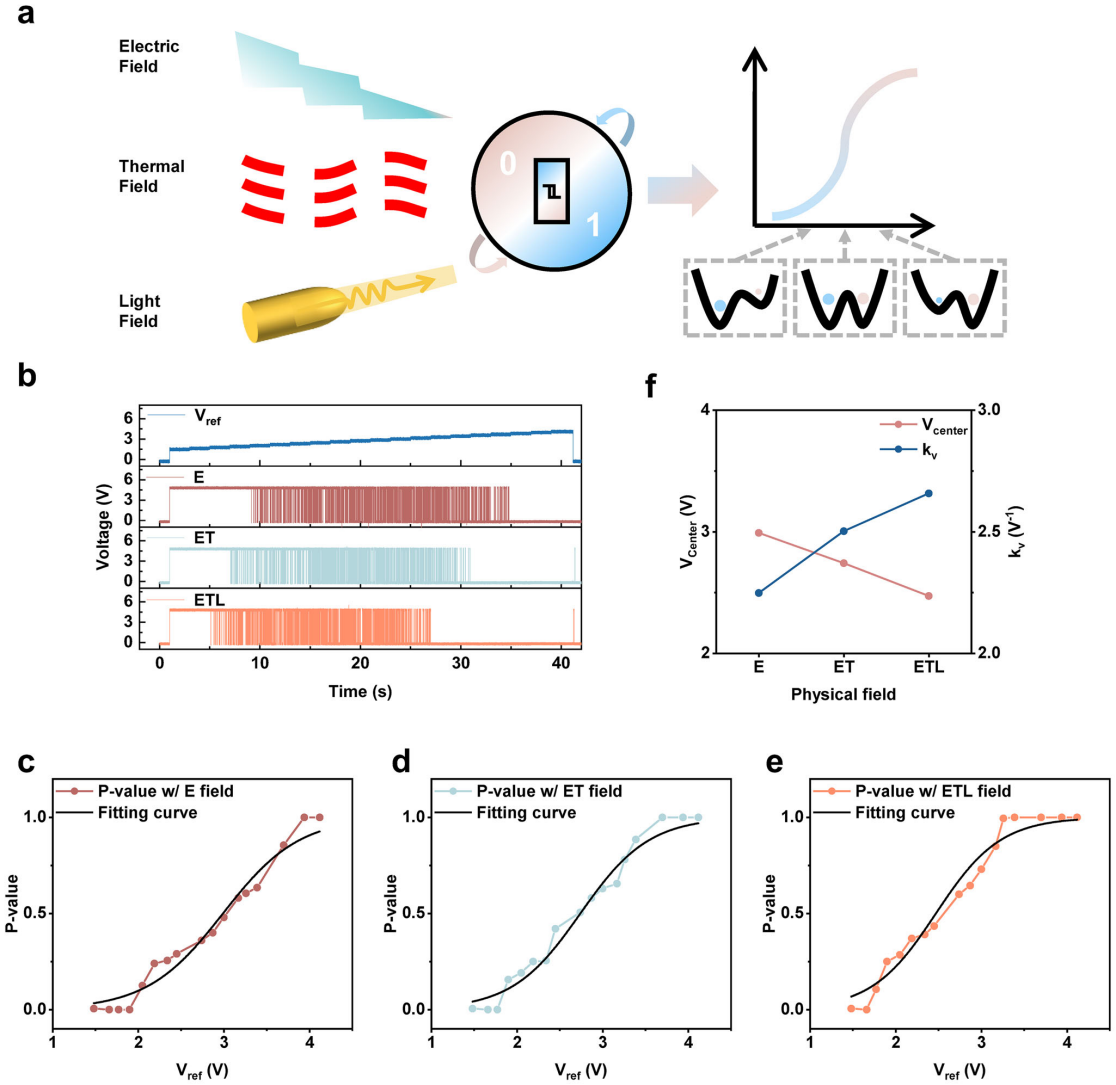
Multi‐physical field modulation of VO_2_ P‐bit. (a) Experimental schematic. The output probability function of a P‐bit device is synergistically controlled through multi‐physical field modulation. (b) Variation in 0/1 trains output by the P‐bit device under different physical fields. (c) to (e) Proportion of 0‐bit output as a function of V_ref_ for applied physical fields E, ET, and ETL, respectively, fitted using a sigmoid function. (f) Variation in sigmoid fitting parameters with different applied physical fields.

Previous studies have shown that the probability of P‐bit outputs can be adjusted between 0 and 1 by tuning the reference voltage V_ref_ [12, 34]. This method can only adjust the V_ref_ within a fixed range, which significantly reduces the device's flexibility. Our current study differs by exploring how interactions with multi‐physical fields influence the P‐bit behavior. This enables the regulation of the probability function within a variable range. Figure 4a depicts the method, where the probability distribution was affected by electric, thermal, and optical fields. In the current study, the effect of multi‐physical field modulation included the following situations: E, electric field applied at room temperature (298 K, no optical excitation); ET, the combination of electric and thermal fields (305 K, no optical excitation); and ETL, which contained the electric, thermal, and optical fields (305 K and 1550 nm pulsed laser irradiation at 10.26 W cm^−^^2^).

Figure 4b presents the resulting output distributions (see Figures S8 and S9). As V_ref_ increases, the proportion of “1” declined while “0” rose, and the presence of additional fields caused a leftward shift in the distributions. To analyze this observation, we measured the proportion of “0” at a fixed V_ref_, and then fitted the data with a Sigmoid probability function (Figure 4c–e) as the following formula:

(1) $$P=\frac{1}{1+e^{-k_{v}\left(V_{\mathrm{ref}}-V_{\mathrm{center}}\right)}}$$

Each point in Figure 4c–e represented probability calculations based on a finite number of random sequences. Due to the memory limitations of the experimental equipment, we used 200 random sequences per point for probability computation. The fitted results were summarized in Figure 4f. It was observed that two main trends emerged: the slope parameter k_v_ increased with the additional fields, while the center value, V_center_ decreased. These shifts reflected the intrinsic response of VO_2_‐based devices under multi‐physical field conditions. The combined use of electric, thermal, and optical fields lowered the threshold voltage V_th_, and shifted the oscillatory region in the phase diagram. This effect propagated through the output circuitry and appeared as a modulation of P‐bit probability. Changes in oscillation amplitude and frequency under multi‐physical field conditions (Figure S7) further supported this interpretation, showing consistency with variations in the Sigmoid parameters. These results demonstrated that synergistic multi‐physical fields allowed effective tuning of the Sigmoid response of P‐bit outputs. Such adjustable probabilistic behavior showed great promise for applications in pattern recognition, combinatorial optimization, and probabilistic computing. To better illustrate the advantages of our device, we have summarized its key distinctions from previous VO_2_‐based P‐bits in Table S1.

The MIT in VO_2_ originates from the complex interplay among electron correlation, lattice distortion, and domain dynamics. While the relationship between electric field effects and Joule heating remains under debate, it is widely acknowledged that thermal activation plays a critical role in the threshold‐induced phase transition. Based on this understanding and our experimental findings, the underlying mechanism can be described as follows: when the device is preconditioned by an optical or thermal field, the influence of the electric field becomes more pronounced, and the resulting phase transition depends on the combined intensity of optical, thermal, and electrical stimuli. When these three mechanisms act in concert, a device that would otherwise fail to form a conductive channel under a sub‐threshold electric field can undergo a sub‐threshold MIT upon simultaneous absorption of external heat or photons. This occurs because the electron correlation strength is perturbed, thereby reducing the energy barrier for conductive filament formation.

Ultimately, this sub‐threshold MIT modulates the initial probability function distribution of the VO_2_‐based P‐bit, enabling the generation of random sequences with distinct probabilistic characteristics under different types and intensities of external physical fields. Moreover, this subthreshold MIT enables the VO_2_ Mott oscillator to operate normally at relatively low voltages, thereby reducing device energy consumption. It is noteworthy that this approach of tuning the probability function via multi‐physical fields is non‐destructive and reconfigurable—the device fully recovers to its initial state once the external fields are removed, an advantage that has not been achieved in previous studies on MIT‐material‐based P‐bit.

### 2.5 SNN Based on VO_2_ Device for Image Recognition

**FIGURE 5:**
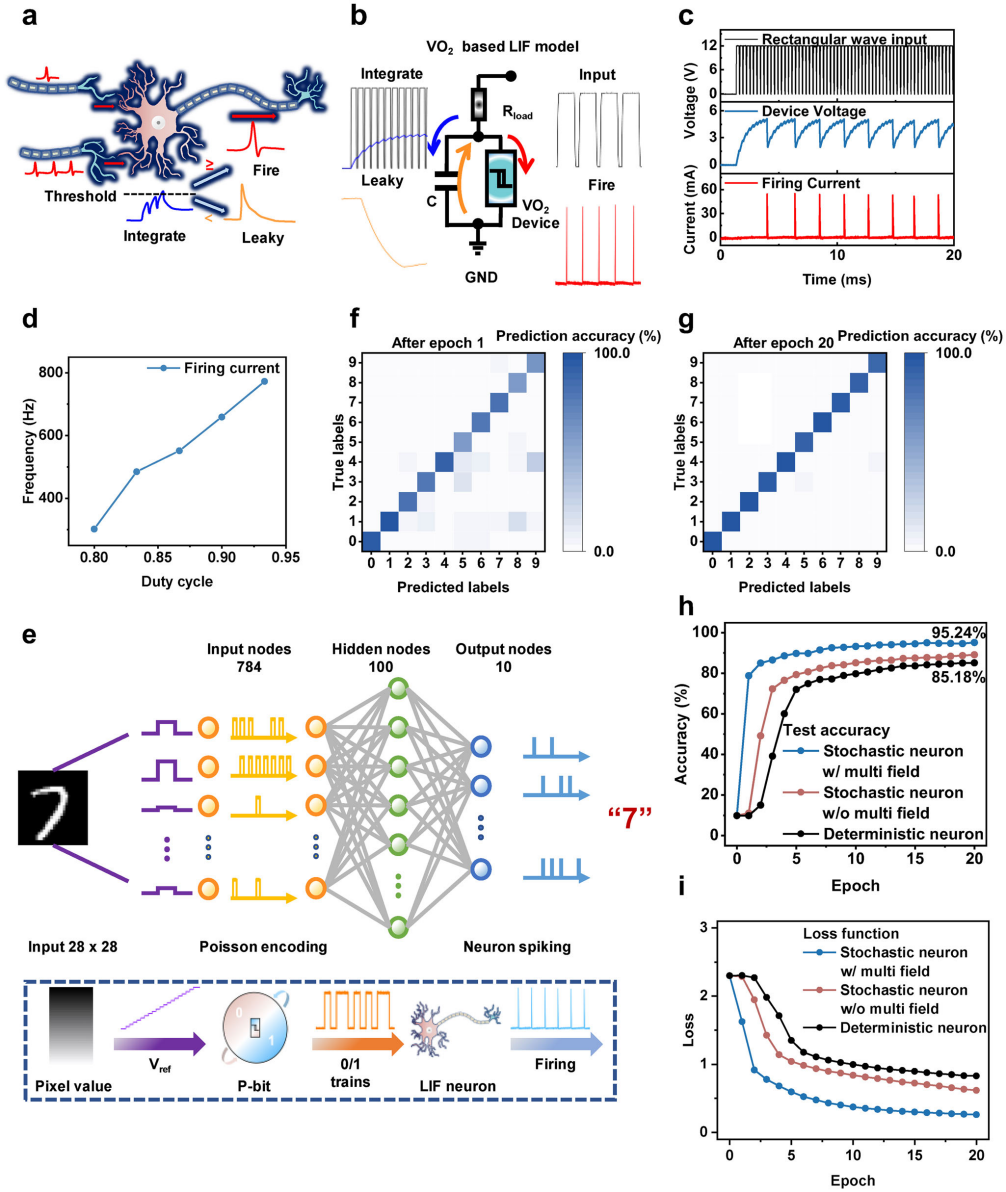
Neuromorphic application based on the VO_2_‐based device. (a) Schematic of a biological neuron. (b) Leaky integrate and fire (LIF) neuron model based on the VO_2_ TS device. (c) Implementation of the VO_2_ LIF model, with input applied as 0/1 voltage pulse trains. (d) Spike firing frequency of the VO_2_ LIF model as a function of the input square wave duty cycle. (e) Schematic of a VO_2_ device‐based SNN simulation for MNIST recognition, consisting of three neuron layers. (f) and (g) Confusion matrices showing recognition accuracy after 1 and 20 training epochs, respectively. (h) and (i) The recognition accuracy of SNN networks with input layers consisting of random neurons (with or without multiphysics modulation) and deterministic neurons, along with the variation of the loss function over training duration.

Research on hardware‐based artificial neural networks has gained significant attention in recent years [10, 36]. To evaluate the potential of the current VO_2_‐based P‐bit devices in such systems, we conducted spiking neural network simulations for MNIST recognition. SNN could use different neuron models, such as Hodgkin–Huxley (HH) [37] or Leaky‐Integrate‐Fire (LIF) [38, 39, 40]. The LIF model worked deterministically with a fixed threshold, while stochastic neurons exhibited random spiking behavior. Since VO_2_ P‐bit devices naturally showed random firing under varying reference voltages, they were suitable for stochastic neuron models [41]. Figure 5a depicts a simplified biological LIF neuron, and Figure 5b–c illustrates the VO_2_ device‐based LIF model, which replicated the standard LIF behavior: input integration, firing, and a refractory period. In Figure 5d, the spiking frequency was observed to increase with input duty cycle (see Figure S11). Building on this, we developed an SNN with stochastic VO_2_ neurons in the input layer and LIF neurons in the hidden and output layers, enabling robust inference despite uncertainty.

Figure 5e illustrates the SNN architecture for MNIST, featuring 784 input neurons, 100 hidden neurons, and 10 output neurons. Each 28 × 28 grayscale image was mapped into reference voltages, with input neurons generating stochastic 0/1 sequences based on pixel intensity. These sequences were integrated by hidden‐layer LIF neurons, which fired when reaching the threshold and passed activity forward. The output neuron with the highest spike count determined the predicted label. The neuron models were directly derived from experimental data. For training, surrogate gradients with a Sigmoid approximation were used for backpropagation [42]. Figure 5f,g displays the confusion matrices after one epoch and after 20 epochs, respectively, with the latter showing clear diagonal dominance, confirming effective feature extraction and classification.

All experimental data required for simulation were collected from VO_2_ devices operating under multi‐physical field modulation. For comparison, simulations using data without multi‐physical field modulation and with a deterministic neural network model in the input layer were also conducted (Figure 5h,i). The multi‐physical field model achieved greater accuracy and faster convergence, due to modified Poisson coding probabilities and altered excitation thresholds in the LIF neurons. The accuracy improvement in SNN simulations after introducing multi‐physical fields primarily stems from optimizing the output characteristics of P‐bits. Experimental data show that with the addition of thermal and optical fields, the probability distribution center of P‐bits shifts to the left, and the slope parameter increases. This indicates that under the same input reference voltage, neurons can generate more discriminative random pulse sequences. Multi‐physical field coupling reduces the phase‐change threshold voltage of VO_2_ devices, enabling input‐layer neurons to more effectively capture subtle features in low‐power inputs and convert them into pulse frequencies. This optimized Poisson coding probability highlights pattern features earlier and more reliably. Due to the lowered excitation threshold from multi‐physical field modulation, output neurons corresponding to correct labels exhibit higher activation frequencies and faster responses. Simulation results informed by experimental device parameters suggested that VO_2_‐based devices under multi‐physical field modulation could improve recognition accuracy and energy efficiency, though hardware implementation remained for future work, multi‐physical.

## 3 Discussion

In this study, we systematically examined VO_2_ based P‐bit device under combined multi‐physical field modulation and explored its potential applications. The relaxation dynamics of the MIT in VO_2_‐based two‐terminal planar devices, showing clear multi‐physical field responses during electrical excitation and excellent cycling stability. Utilizing their self‐sustained oscillations, we then built a P‐bit device whose output probability could be actively adjusted by electric, thermal, and optical stimuli. Compared to traditional P‐bits, this design provided greater flexibility in probability control, with multi‐physical field regulation adding an extra degree of freedom that was advantageous for applications like information encryption and probabilistic computing. Lastly, simulations of a three‐layer spiking neural network achieved 95.14% accuracy on MNIST digit classification, demonstrating the strong potential of these devices for neuromorphic computing hardware.

In summary, VO_2_ P‐bit devices controlled by synergistic multi‐physical fields would possess broad prospects in energy‐efficient computing, pattern recognition, and information security. This research not only offered new insights into VO_2_‐based devices but also underscored the importance of phase‐change materials in multi‐physical field coupling for advanced device architectures.

## 4 Methods

### 4.1 Film Deposition

About 50 nm VO_2_ thin films were grown on C‐cut sapphire substrates by using rf assisted oxide molecular beam epitaxy (O‐MBE) technique. During the film deposition process, the substrate temperature and oxygen partial pressure were maintained at 550°C and 2 × 10^−3^ Pa, respectively. The deposition process was monitored in situ using reflection high‐energy electron diffraction (RHEED). Crystal oscillator (Inficon SQM‐160) was adopted to measure the film deposition rate.

### 4.2 Device Fabrication

Standard UV photo‐lithography processes were used to deposit electrodes on the prepared VO_2_ thin films. Specifically, the thin film samples, which had been ultrasonically cleaned, were first spin‐coated with positive photoresist SPR955‐0.7 (3000 rpm, 40 s) and then baked at 100°C for 90 s. Then the spin‐coated samples were exposed using UV lithography (Karl Suss, MABA6), followed by development with AZ300MIF solution for 50 s. After that, the patterned samples were deposited with a 20 nm Ti/80 nm Au electrode using electron beam thermal evaporation (Lesker, Lab18). The samples with the deposited electrodes were heated in an 80°C NMP water bath for 2 h for lift‐off. Finally, the micro/nano‐fabricated devices were surface‐cleaned using radiofrequency oxygen plasma (PLUTOVAC, PLUTO‐30).

### 4.3 Material Characterizations

The crystal structures of the prepared VO_2_/Al_2_O_3_ thin films were characterized by using an X‐ray diffractometer (XRD, model X'Pert MPD, Philips) with Cu Kα radiation (λ = 1.54178 Å). Raman spectra at different temperatures were obtained using a HORIBA LabRAM HR Raman system, with an excitation wavelength of 532 nm and a laser power of 5 mW. X‐ray photoelectron spectroscopy (XPS, Kratos, AXIS Supra+) was performed using Al Kα X‐rays at a base vacuum of 5.0 × 10^−10^ Torr, with a photon energy of 1486.6 eV. X‐ray absorption near‐edge structure (XANES) measurements were conducted at the XMCD beamline (BL12B) of the National Synchrotron Radiation Laboratory (NSRL) in Hefei. The total electron yield (TEY) mode was used to collect the sample drain current under a vacuum better than 3.5 × 10^−9^ Torr. The energy range was 100–1000 eV, with an energy resolution of 0.2 eV. The ultraviolet‐visible‐near‐infrared spectroscopy (Hitachi UH5700) was used to measure the light transmission spectrum of the heater under different temperatures and voltage inputs. The cross‐section and surface morphology of the film were characterized at room temperature using SEM (SU8220, Hitachi). The surface morphology and structure of the VO_2_ films were characterized using atomic force microscopy (AFM, MFP‐3D‐Origin).

### 4.4 Electronic Transport Measurement

The electrical measurements of the VO_2_ based device were performed at room temperature using a semiconductor parameter analyzer (FS‐Pro) in conjunction with a Semi‐share four‐probe station. After bonding, the device was connected as an independent circuit component to a commercial breadboard, along with DIP‐packaged circuit components (such as SN74LS273N and NE5532P) and an oscilloscope, to complete subsequent testing. The testing process utilized a Keithley 2450 Source‐meter for regulated power supply.

### 4.5 Spiking Neural Network Simulations

The three‐layer spiking neural network with an architecture of 784 × 100 × 10 was simulated in Python for MNIST digit classification, where the stochastic neurons and LIF neurons were implemented by the VO_2_ P‐bit and VO_2_ two‐terminal planar devices, respectively. Input spike trains were propagated through the network over 50 discrete time steps. Hidden‐layer outputs were linearly transformed and passed to the output LIF layer, which integrated excitatory and inhibitory currents, updated membrane potentials, and emitted spikes when potentials exceeded the adaptive threshold. Surrogate‐gradient backpropagation with a sigmoid approximation enabled gradient computation through the non‐differentiable spike function. Performance was evaluated using classification accuracy and confusion matrices. All experiments used PyTorch 1.12.1 with a batch size of 128.

## Author Contributions

C.W.Z. and B.W.S. conceived the research. C.W.Z. supervised the project. C.W.Z., B.W.S., and J.L.Z. designed the experiments. B.W.S. and J.J.L. performed the sample fabrication and device measurement. B.W.S. performed the SNN simulations. T.Z., M.L.L., Z.H.L., C.W., C.Y.L., Y.X.C., and X.K.H. contributed to the data analysis. B.W.S. and T.Z. prepared the manuscript, with input and corrections from all authors. All authors analyzed the results and implications and commented on the manuscript at all stages.

## Conflicts of Interest

The authors declare no conflicts of interest.

## Supporting information




**Supporting File**: advs74458‐sup‐0001‐SuppMat.docx.

## Data Availability

The data that support the findings of this study are available from the corresponding author upon reasonable request.
