# Supplementary material for: Multi‐Physical Field Modulated P‐Bit Device Based on VO2 Thin Film
Source: Adv Sci (Weinh). 2026 Feb 16;13(24):e24248. doi: 10.1002/advs.202524248 (PMC13115954; doi:10.1002/advs.202524248)
Supplement: Supplementary file 1 — Supporting File: advs74458‐sup‐0001‐SuppMat.docx. [file ADVS-13-e24248-s001.docx]

**Supplementary Information for**

**Multiphysical Field Modulated P-bit Device Based on VO_2_ Thin Film**

Bowen Sun^1^, Jianjun Li^1^, Ting Zhou^1^, Jinglin Zhu^1^, Meiling Liu^1^, Zhihan Lin^1^, Chang Wang^1^, Chengyu Li^1^, Yingxue Chen^1^, Xiaokun Huo^1^, Chongwen Zou^1*^

^1^ National Synchrotron Radiation Laboratory, School of Nuclear Science and Technology, University of Science and Technology of China, Hefei, Anhui 230029, P. R. China

*Corresponding Author: [czou@ustc.edu.cn](mailto:czou@ustc.edu.cn)

**Contents:**

**Supplementary Figures:**

**Figure S1-S11**

**Table S1**

**Reference**

**Supplementary Figures:**

**
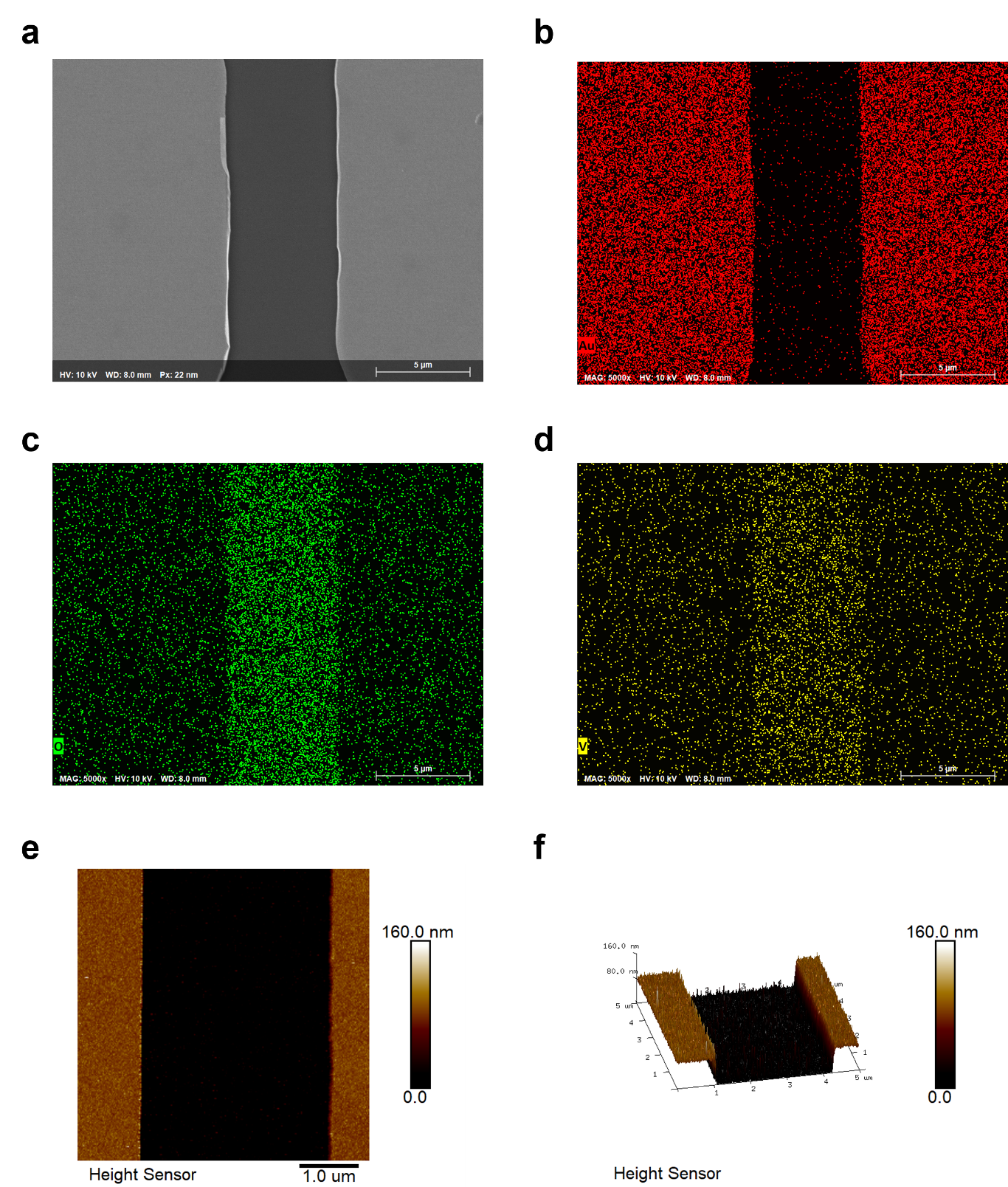
**

**Figure S1.** Characterizations of the conductive channel gap in two-terminal VO_2_ device. a)SEM image for the VO_2_ gap. b-d) EDS element mapping. e,f) AFM 3D morphologies.

**
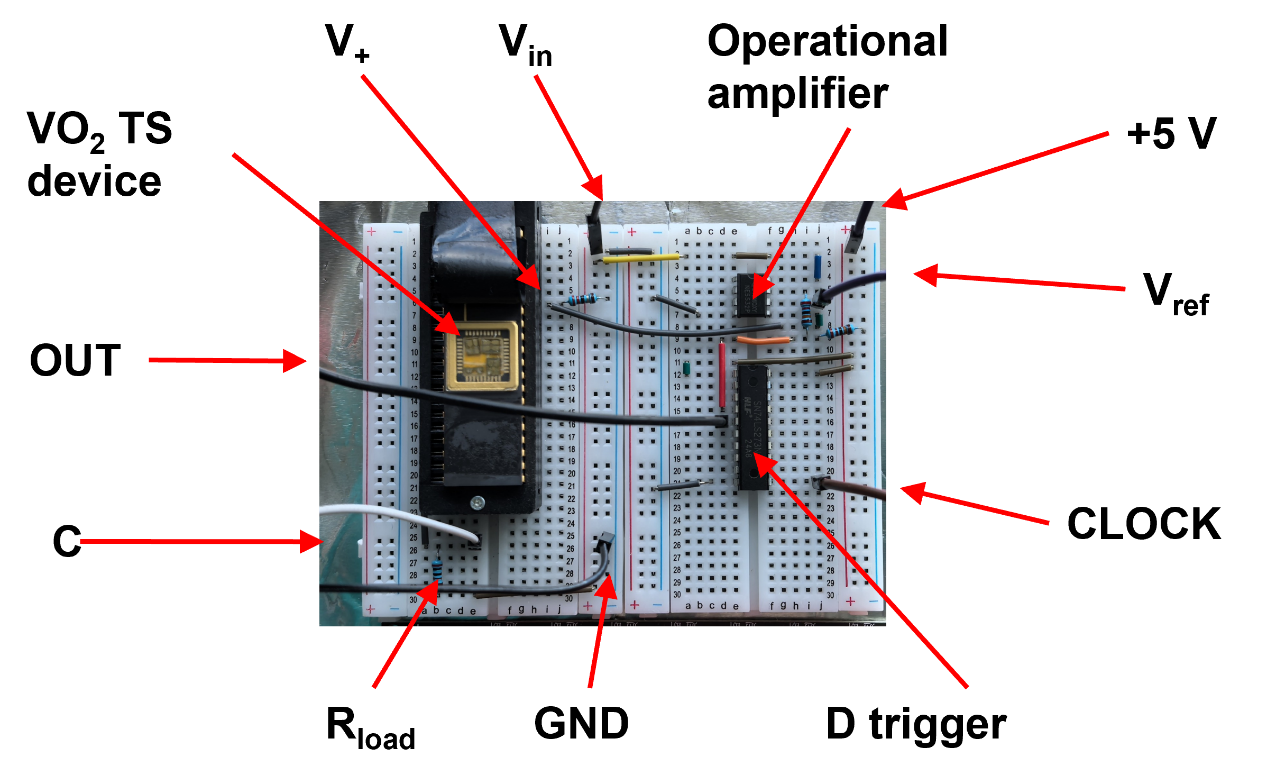
**

**Figure S2.** The optical image for the fabricated P-bit circuit integrated into a breadboard.


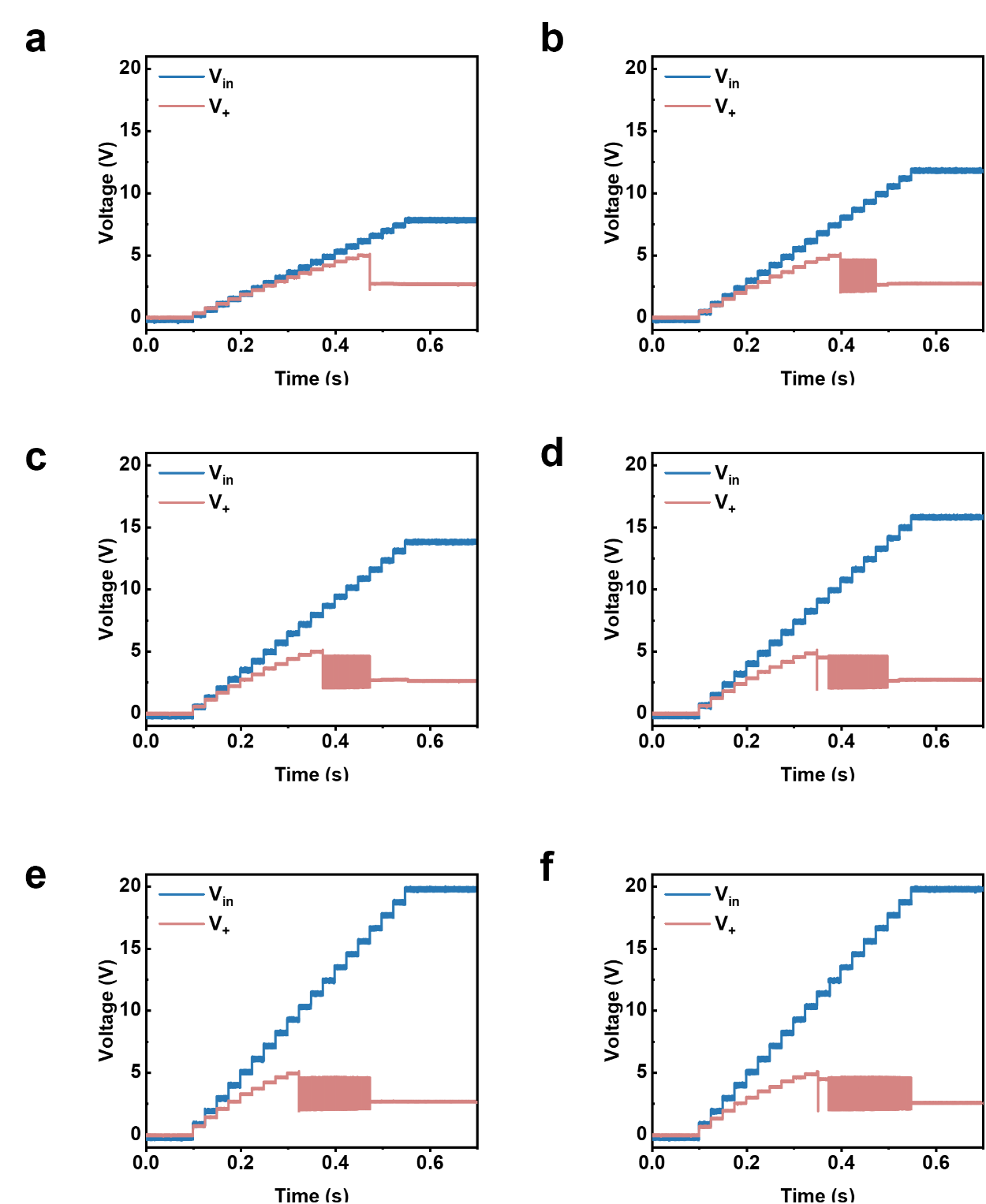


**Figure S3.** Step voltage scan for the VO_2_ Mott oscillator under different load resistances. a-f) 500/1000/1200/1500/1800/2300 ohm.

**
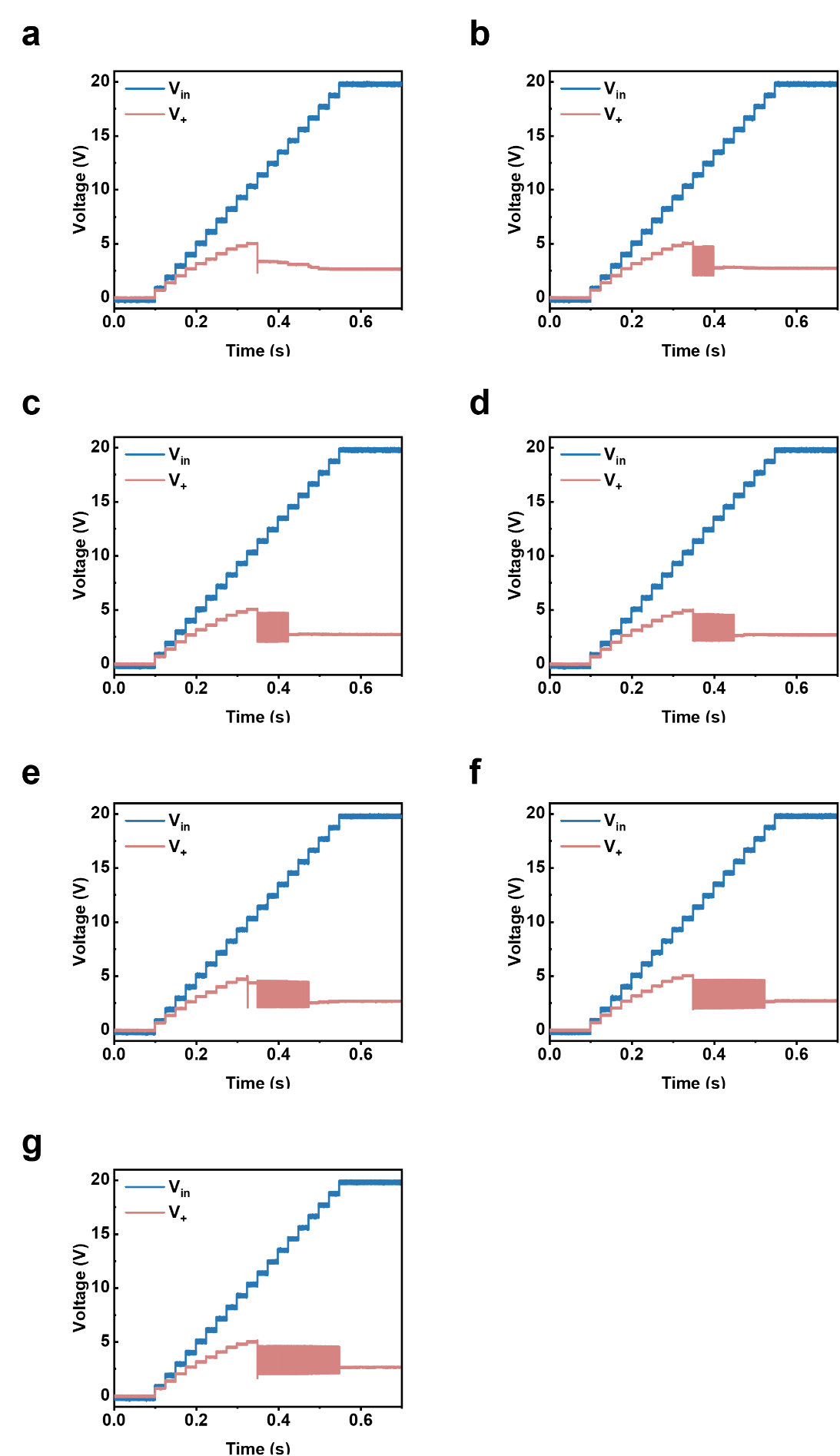
**

**Figure S4.** Step voltage scan for the VO_2_ Mott oscillator under different parallel capacitors. a-g) 1/3/5/10/20/50/70 nF.

**
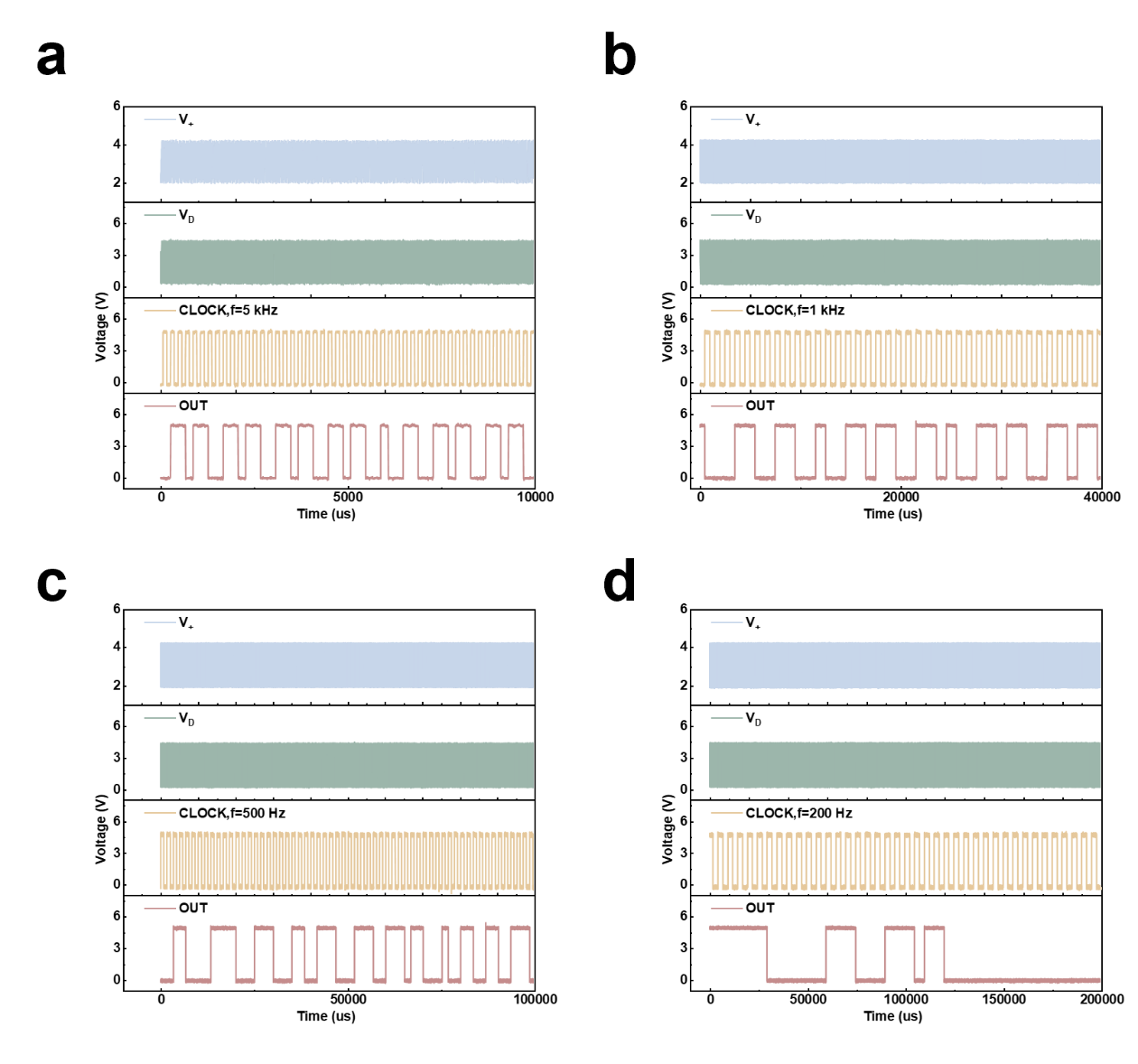
**

**Figure S5.** The bit output of the circuit. a–d) Clock signal frequencies are 5 kHz, 1 kHz, 500 Hz, and 200 Hz, respectively.


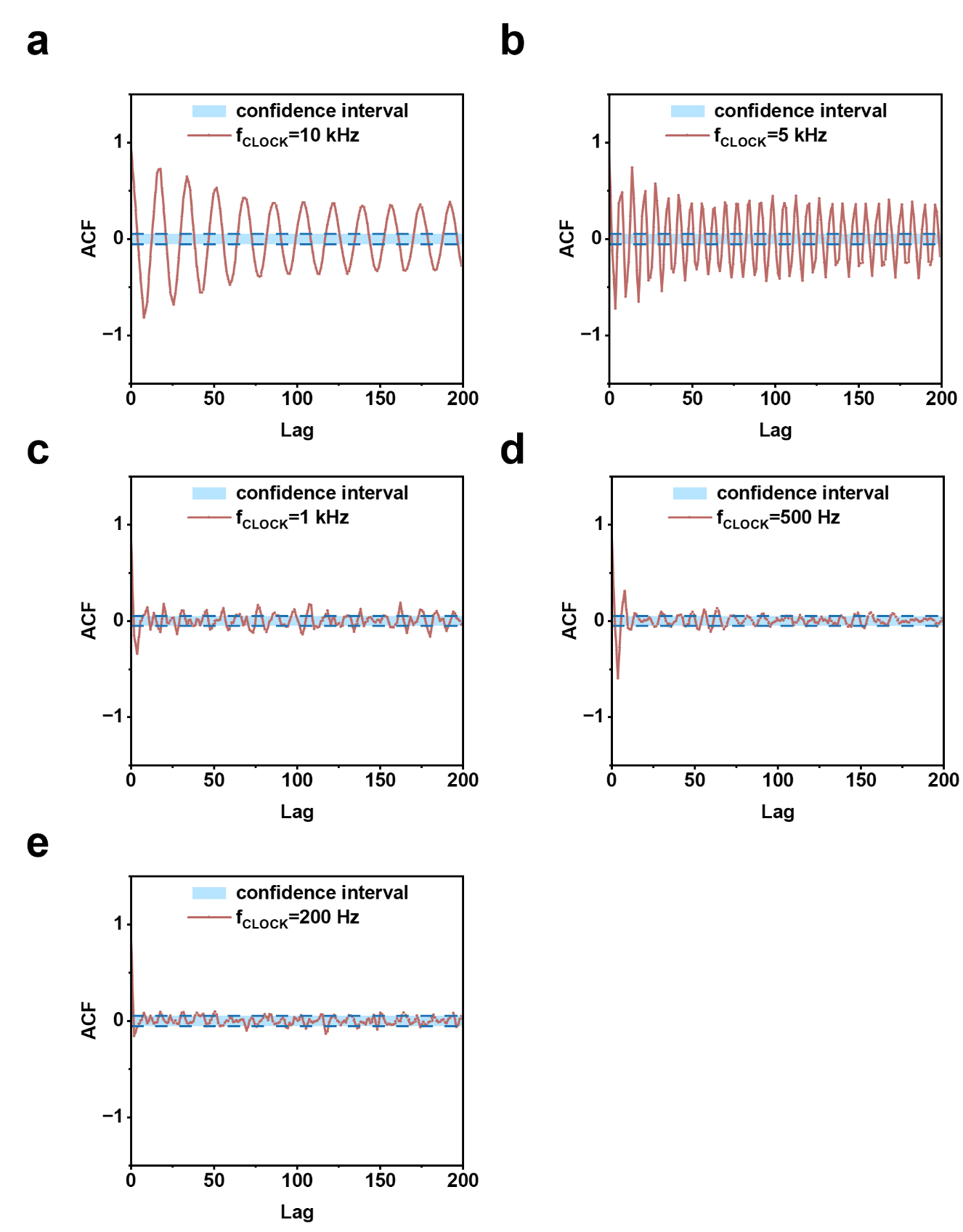


**Figure S6.** The autocorrelation coefficient of bit trains, with a 95% confidence interval. a–e) Clock signal frequencies of 10 k/5 k/1 k/500/200.


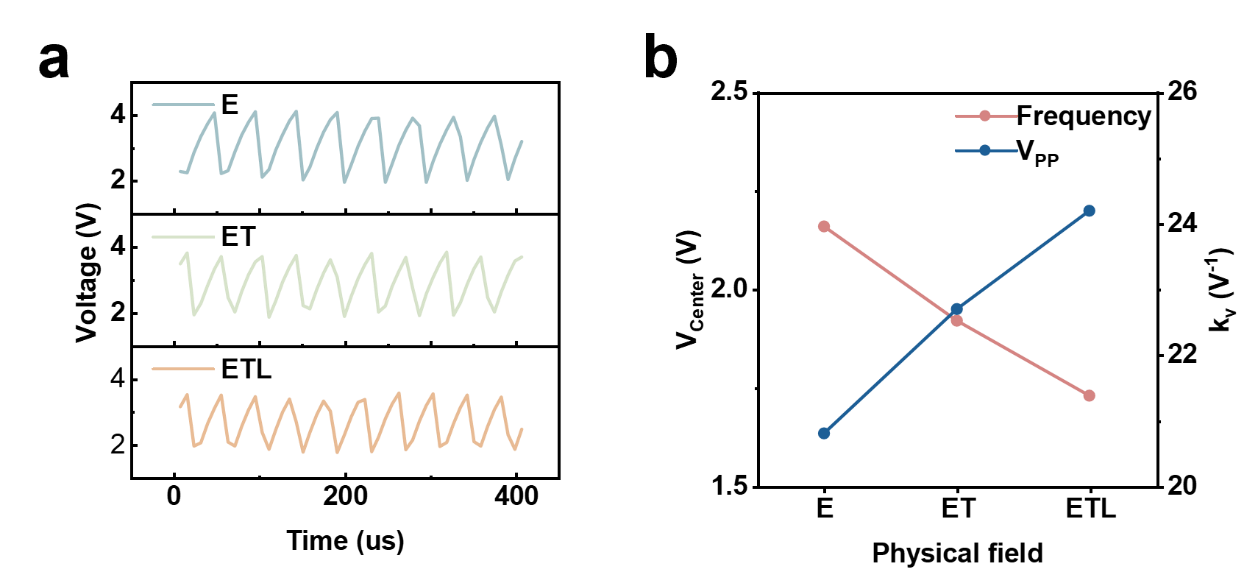


**Figure S7.** The multi-physics modulated oscillatory waveforms.


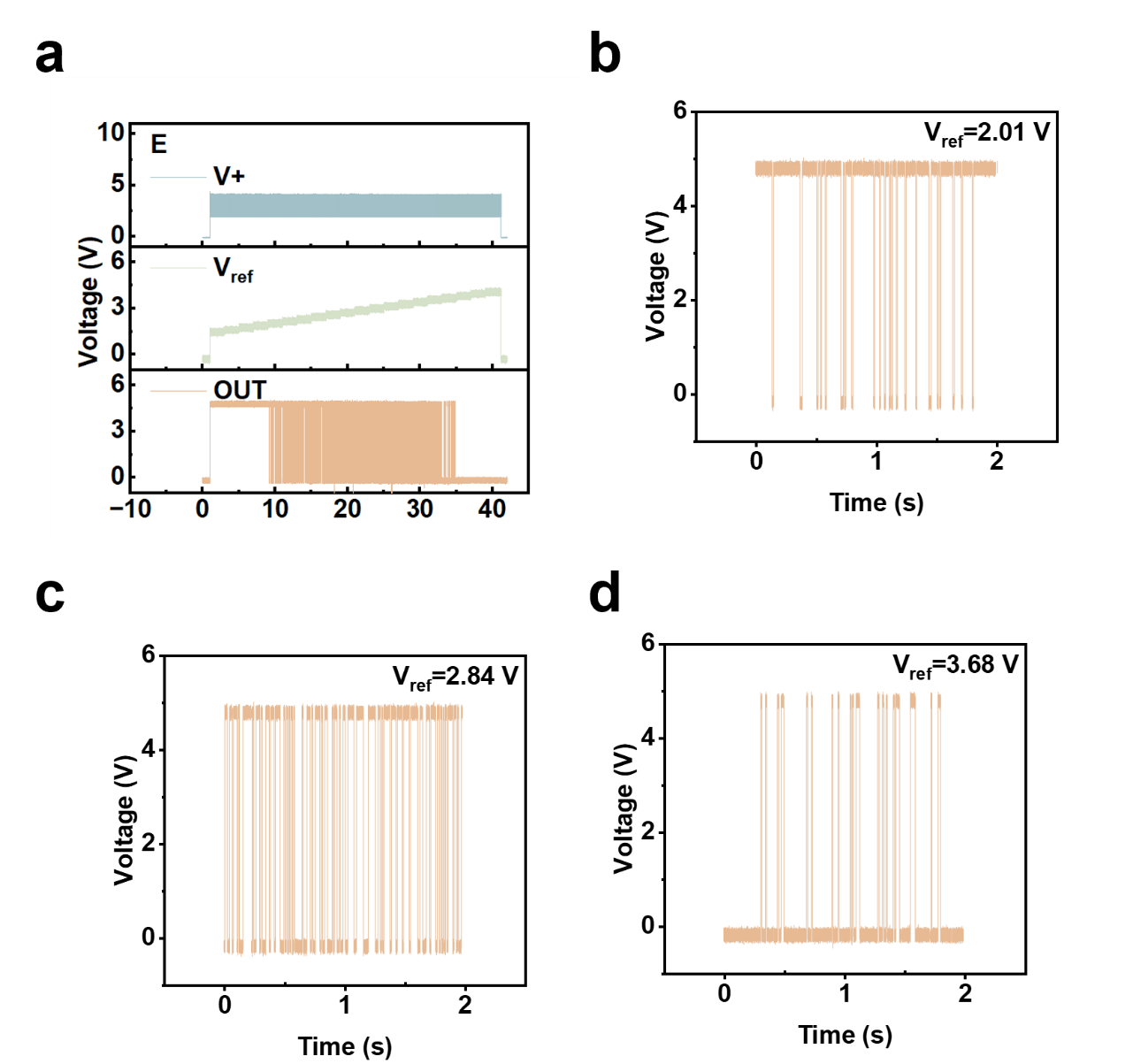


**Figure S8.** Under the influence of E condition (electric field), the output 01 trains of the P-bit device. a) Overall output during voltage mapping scan. b-d) V_ref_: 2.01 V/2.84 V/3.68 V.


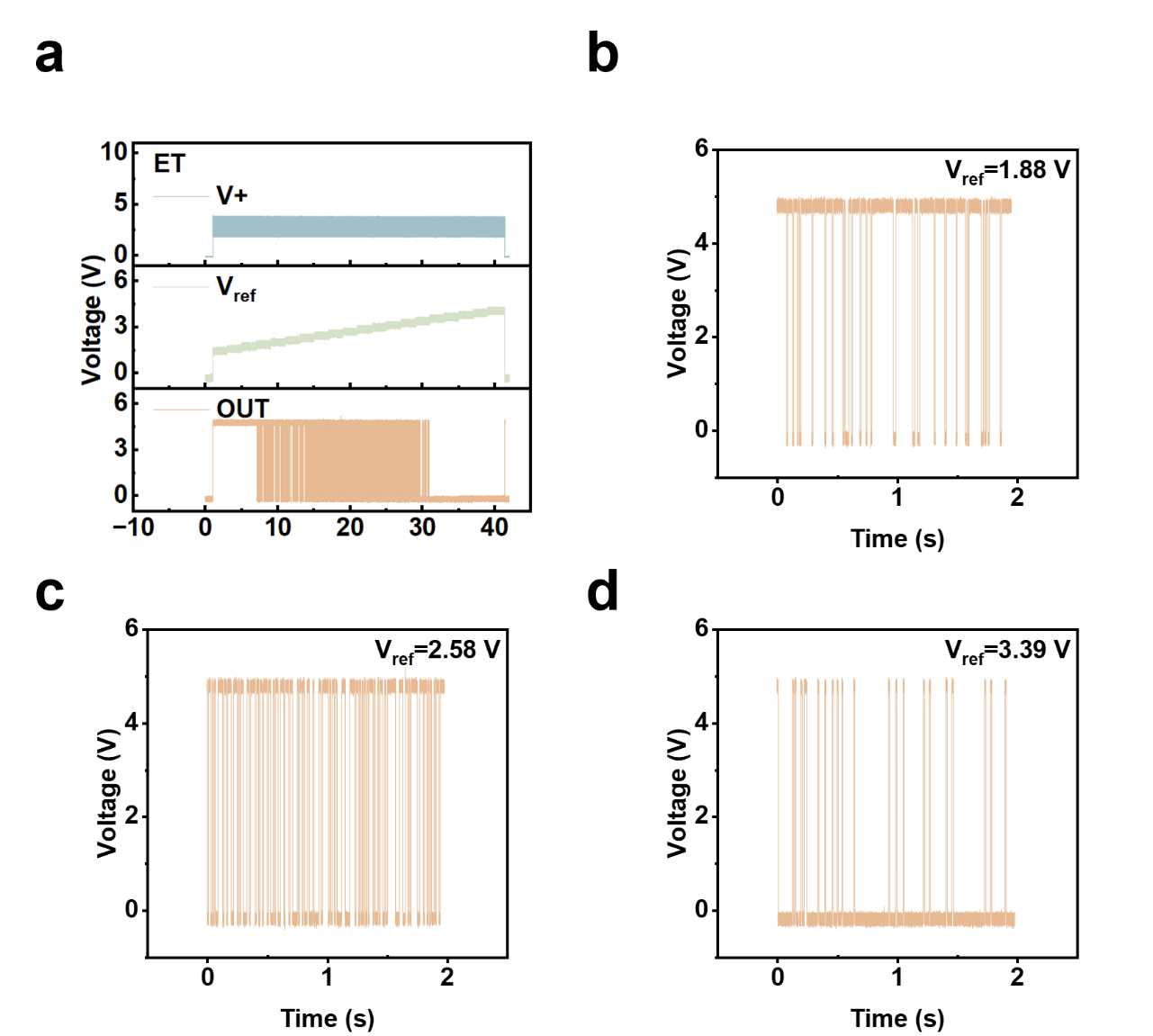


**Figure S9.** Under the influence of ET condition (Electric field and temperature field), the output 01 trains of the P-bit device. a) Overall output during voltage mapping scan. b-d) V_ref_: 1.88 V/2.58 V/3.39 V.


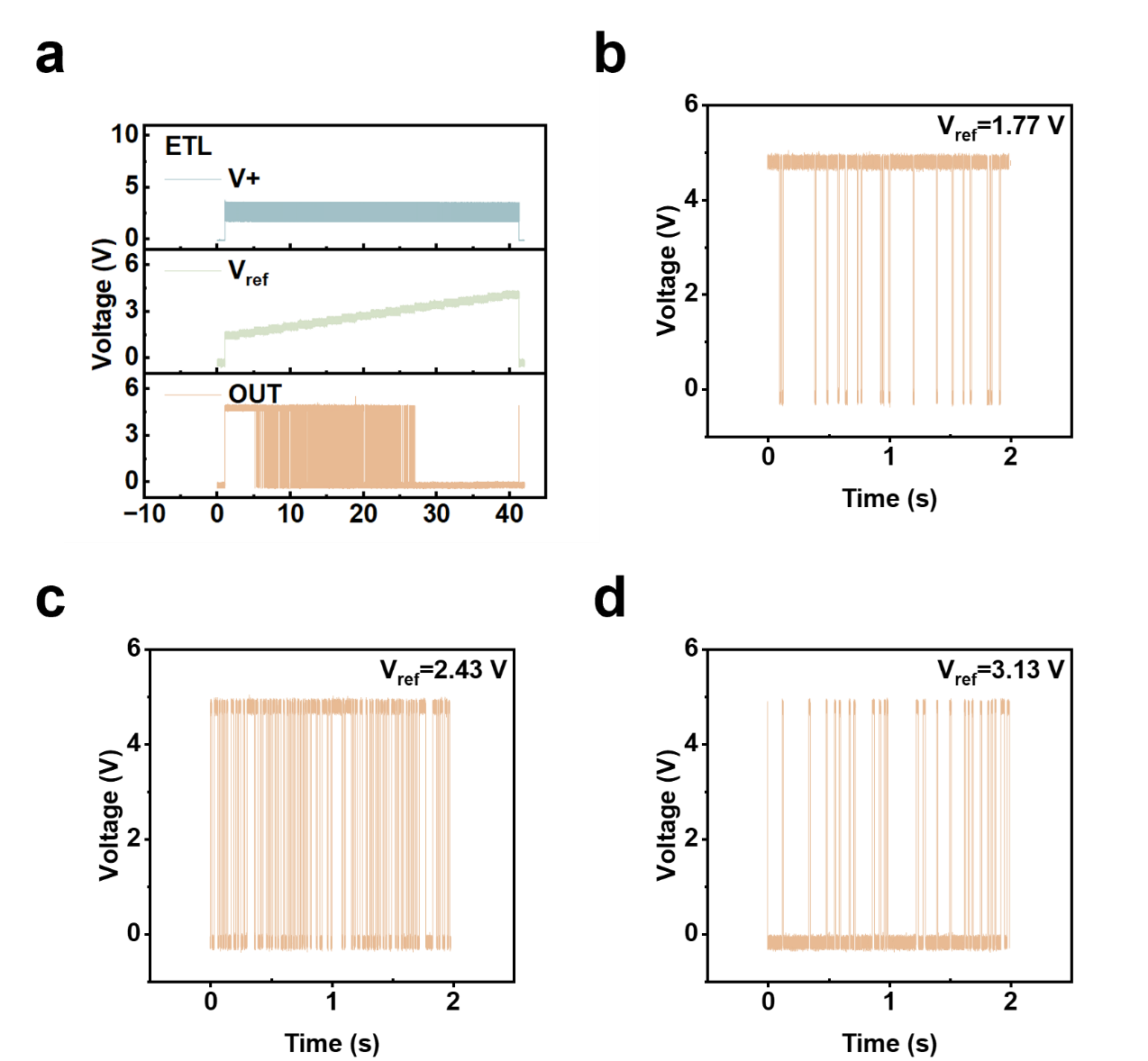


**Figure S10.** Under the influence of ETL condition (Electric field, temperature field and laser radiation), the output 01 trains of the P-bit device. a) Overall output during voltage mapping scan. b-d) V_ref_: 1.77 V/2.43 V/3.13 V.


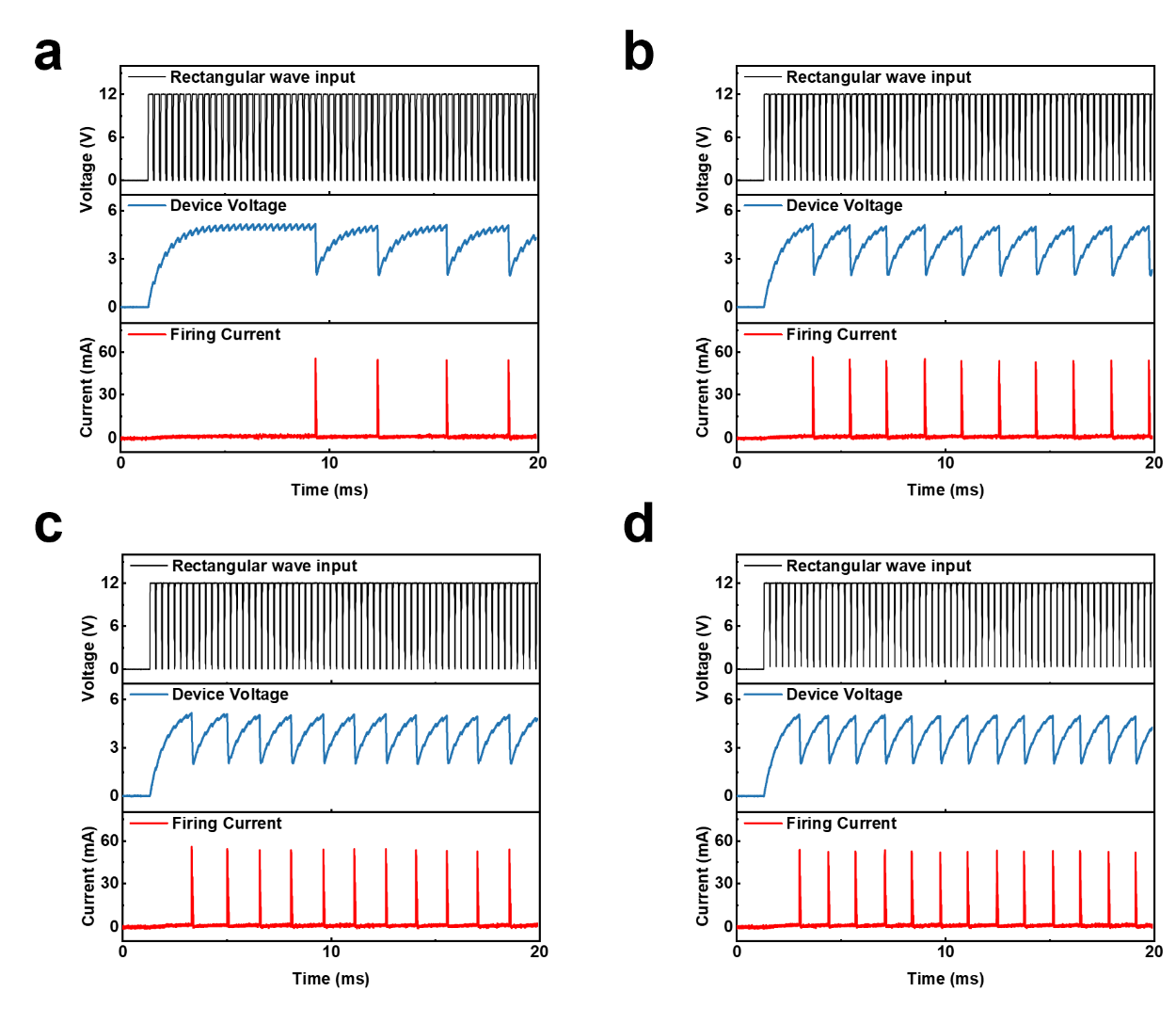


**Figure S11.** The VO_2_ LIF model for different conditions: a–d) Input voltage duty cycles of 0.8/0.867/0.9/0.933.

| Device Structure | Source of Randomness | Control Mechanism | Tunable & Reconfigurable | Tuning Range (Kv/V⁻¹) | Endurance |
| --- | --- | --- | --- | --- | --- |
| Au/VO₂/Au,  ^1^ | Subthreshold firing | Electric field | No | None | >4.9×10⁴ |
| TiN/VO₂/Pt,  ^2^ | Subthreshold firing | Electric field, Pt NP doping | Yes, not reconfigurable | 25–160 | >1.3×10⁵ |
| Pd/VO₂/Au,  ^3^ | Subthreshold firing | Electric field, H doping | Yes, not reconfigurable | 25–160 | >1×10⁴ |
| Pt/NbOₓ/Pt,  ^4^ | Mott oscillator | Electric field | No | None | >4×10⁸ |
| Au/VO₂/Au,  This work | Mott oscillator | Electric, thermal, and optical fields | Yes, reconfigurable | 2.2–2.6 | >1×10⁶ |

**Table S1**: Comparison with existing works on metal-insulator transition material-based P-bits.

**Reference：**

(1) del Valle, J.; Salev, P.; Gariglio, S.; Kalcheim, Y.; Schuller, I. K.; Triscone, J.-M. Generation of Tunable Stochastic Sequences Using the Insulator-Metal Transition. *Nano Letters* **2022**, *22* (3), 1251-1256, Article. DOI: 10.1021/acs.nanolett.1c04404.

(2) Seo, Y.; Park, Y.; Hur, P.; Jo, M.; Heo, J.; Choi, B. J.; Son, J. Promotion of Probabilistic Bit Generation in Mott Devices by Embedded Metal Nanoparticles. *Advanced Materials* **2024**, *36* (31). DOI: 10.1002/adma.202402490.

(3) Deng, S. B.; Park, T. J.; Yu, H. M.; Saha, A.; Islam, A.; Wang, Q.; Sengupta, A.; Ramanathan, S. Hydrogenated VO<sub>2</sub> Bits for Probabilistic Computing. *Ieee Electron Device Letters* **2023**, *44* (10), 1776-1779. DOI: 10.1109/led.2023.3303875.

(4) Kim, G.; In, J. H.; Kim, Y. S.; Rhee, H.; Park, W.; Song, H.; Park, J.; Kim, K. M. Self-clocking fast and variation tolerant true random number generator based on a stochastic mott memristor. *Nature Communications* **2021**, *12* (1), Article. DOI: 10.1038/s41467-021-23184-y.
